# Competent Psychopharmacology

**DOI:** 10.1177/070674371405900802

**Published:** 2014-08

**Authors:** David M Gardner

**Affiliations:** 1 Professor, Department of Psychiatry and College of Pharmacy, Dalhousie University, Halifax, Nova Scotia.

**Keywords:** psychotropics, competency, prescribing, psychiatry, mental illness, antipsychotic, antidepressant, post-graduate education, continuing education, patient experience

## Abstract

There is little doubt that undergraduate and post-graduate training of physicians, pharmacists, and nurses is insufficient to prepare them to use psychotropics safely and effectively, especially in the context of their expanded off-label uses. Therefore, the development of competencies in psychotropic prescribing needs to be approached as a long-term, practice-based learning commitment. Proposed are the abilities and knowledge components necessary for safe and effective use of psychotropics. Typical challenges in prescribing for chronic and recurrent illnesses include highly variable responses and tolerability, drug interactions, and adverse effects that can be serious, irreversible, and even fatal. Prescribing psychotropics is further complicated by negative public and professional reports and growing patient concerns about the quality of care, and questions about the efficacy, safety, and addictive risks of psychotropics. Increased efforts are needed to enhance clinical training and knowledge in psychopharmacology among trainees and practising clinicians, with more comprehensive and sustained attention to the assessment of individual patients, and greater reliance on patient education and collaboration. Improved competence in psychotropic prescribing should lead to more informed, thoughtful, and better-targeted applications as one component of more comprehensive clinical care.

## Abstract (fr)

Il y a peu de doutes que la formation de premier cycle et postdoctorale des médecins, des pharmaciens et des infirmiers ne suffit pas à les préparer à utiliser les psychotropes de façon sécuritaire et efficace, surtout dans le contexte de l’utilisation non indiquée (off label) largement répandue. Par conséquent, l’acquisition d’aptitudes à prescrire des psychotropes doit être considérée comme un engagement à long terme d’apprentissage, basé sur la pratique. Nous proposons les capacités et les composantes des connaissances nécessaires à une utilisation sécuritaire et efficace des psychotropes. Les problèmes typiques liés à la prescription pour des maladies chroniques et récurrentes sont notamment des réponses et une tolérabilité extrêmement variables, des interactions de médicaments, et des effets indésirables qui peuvent être sérieux, irréversibles, même fatals. Prescrire des psychotropes est compliqué en plus par des articles pour le public et les professionnels négatifs, et par les préoccupations croissantes des patients au sujet de la qualité des soins, ainsi que par les questions sur l’efficacité, l’innocuité, et les risques de dépendance des psychotropes. Il faut plus d’efforts pour améliorer la formation et les connaissances cliniques en psychopharmacologie chez les stagiaires et les cliniciens qui pratiquent, en portant une attention plus minutieuse et soutenue à l’évaluation des patients individuels, et en se reposant davantage sur l’éducation et la collaboration des patients. De meilleures compétences pour prescrire des psychotropes devraient entraîner des applications plus éclairées, réfléchies et mieux ciblées, comme composantes de soins cliniques plus complets.

Neuropharmacologists continue to seek the next giant step forward in the medication-based treatment of mental illness. However, the decades-long absence of truly novel advances underscores the difficulty of a rational approach to therapeutic innovation in psychiatry.^1^ For now, we need to pursue optimal use of available psychotropics and investigations of pressing clinical questions. These include clarifying for particular types of patients which psychotropics provide the highest probability of avoiding hospitalization and minimizing illness burden, as well as supporting stable, loving relationships, long-term employment, personal achievement, and maximal life expectancy.

It is important, first, to recognize that most psychotropic prescriptions do not involve a psychiatrist, at least not directly. Primary care clinicians account for most use of antidepressants, benzodiazepine sedatives, and antipsychotics, with significant expansions into geriatric and pediatric care in recent years. This expanded adoption of psychotropic treatments outside of psychiatry is, in part, due to the greater safety of newer agents in overdose. However, this expanding usage has had notable, unintended consequences for the quality of patient care and for education and training of prescribing clinicians.

In the 1980s, when I first started practicing as a pharmacist in psychiatry, the leading therapies were TCAs, benzodiazepines, carbamazepine, lithium, and neuroleptics. Owing to concern about potentially serious adverse events, treatment rejection, unsatisfactory treatment response, and lethality in overdose, comprehensive initial clinical assessment and vigilant follow-up—with ongoing evaluations of response, tolerance, and adherence—were the order of the day. That day was soon to end. Newer psychotropics, with wider therapeutic indexes (margins of safety) and notably different adverse effect profiles (including modern antidepressants and antipsychotics), are much more readily accepted by prescribing clinicians of all stripes, and evidently considered safe enough as to encourage lowered clinical vigilance. Unlike 25 years ago, today patients with a lower burden of mental health problems and illness severity are being prescribed newer antidepressants, anticonvulsants with mood stabilizing properties, and modern antipsychotics.^2^ This change of practice appears to involve remarkably little consideration of reasonable alternatives—pharmacological and nonpharmacological. Prior to the introduction of fluoxetine in 1989, depressed patients typically were prescribed a TCA, with an initial prescription for 7 or 14 days to allow for assessing adverse effects and responses, to limit the risk for self-harm in the event of an overdose, and to limit waste owing to poorly tolerated treatments. In essence, a medication’s potential for toxicity demanded thoughtful and attentive clinical care.

Today, even a first prescription for a modern antidepressant is almost always for 30 days, with few, if any, planned follow-up visits.^3^,^4^ Safer medicines have encouraged major changes that, arguably, represent a degradation in the quality of clinical care and prompt the question of whether patient outcomes have advanced or regressed as a result.^2^,^5^ Clearly, the issue is not with the newer medicines, but rather with those of us who prescribe, model, educate, promote, dispense, or take them.

## Training

In principle, psychiatrists are the most expert of mental health professionals in the optimal clinical application of psychopharmacological treatments. Training for this specialty involves learning about psychotropics, first as an undergraduate medical student during introductory courses on neuroscience, pharmacology, and the brain and behaviour, and a typically brief clinical psychiatric clerkship. These rudimentary components of medical training apply to all future physicians, who will regularly encounter the 4% or so of the general population with a serious mental illness, whose treatment almost always involves 1 or more psychotropics.^6^ Such basic medical training is far from adequate for competence in the safe and effective use of psychotropics in everyday clinical practice.

As a classic duality, psychotropics have the potential not only to support major amelioration and occasional recovery from mental illnesses but also to risk causing serious adverse effects of a magnitude that dwarves the reason for the prescription.^7^,^8^ As such, I propose that matters summarized in Table 1 are basic and essential to assuring safe and effective use of psychotropics. There is little doubt that undergraduate training of physicians, surgeons, pharmacists, physician assistants, nurse practitioners, and nurses is insufficient to prepare them to use psychotropics safely and effectively, or, indeed, to gain an adequate understanding of psychiatric illnesses. Therefore, postgraduate training and practice-based learning are of critical importance.

Psychopharmacology education during the several years of psychiatric residency is mostly experience-based, with individual patients and variable levels of intensity of clinical supervision by senior colleagues, supplemented by lectures, seminars, and journal clubs, along with, what appears to be, declining emphasis on comprehensive case-review conferences. Less specialized training programs for primary care physicians, and others, involve far less training in the evaluation and treatment of people living with mental illness and in the informed use of psychotropics. The quality of such training experiences can vary widely among and even within training programs. Most of the variability rests on the teacher and the learner.

**Highlights**

- Training in the safe and effective use of psychotropics is insufficient and merits review at the undergraduate, post-graduate, and continuing professional development levels.
- Advances in training and education about psychotropics need to challenge current trends, on the grounds of insufficient safety and efficacy data, and on clinical applications contrary to the available evidence.
- Use of psychotropics is expected to be more selective and thoughtful as competencies improve and alternative interventions are explored more earnestly.

I did not know it at the time, but my first positions in mental health aligned me with some outstanding psychiatric clinicians and educators. Since then, I have seen a wide range of clinical training supervisors of psychiatric residents, from those who neglect to encourage residents to learn about and use psychotropics conscientiously, to those who are both gifted teachers and motivators and virtual warehousers of knowledge, and sometimes involved in research in psychopharmacology. I watched supervisors who were most impressive take time with their residents to discuss the care of particular patients and thoughtfully examine pharmacological management in the context of comprehensive clinical assessment and care. Residents were given opportunities to demonstrate their knowledge and to deepen it, often by having specific, patient-pertinent pharmacological questions to be discussed at a next meeting after some reading. This approach was invaluable to my own learning. The better supervisors articulated their diagnostic considerations and linked them with treatment options, thinking aloud about reasons for and against each, and acknowledging where their knowledge or the evidence had gaps. Their overt modelling of how to think about and use psychotropics in the clinical care of patients with serious mental illness had an enduring effect on me and, I believe, on the psychiatric residents they supervised. It was in the past, and remains, a major challenge, even for outstanding training programs, to establish and maintain such high-quality training consistently, across sites, within individual departments or institutions.

While clinical training supervisors continue to play a major role, much of the onus of becoming competent and well-informed is on the trainee. Ask a few experienced psychiatrists at tertiary teaching hospitals and you will be sure to hear about a slacker attitude of this generation’s learners. However, did their supervisors say the same about them when they were learning how to safely prescribe the Newcastle cocktail (phenelzine, lithium, and L-tryptophan) without causing the yet-to-be-labelled, potentially fatal serotonin syndrome?^9^ Is this merely recall bias influenced by collegial alliances? Did they prefer reading the original research after extensively leafing through *Index Medicus* or did they take the early editions of *Kaplan and Sadock’s*^10^ at face value?

Necessary components for effective self-directed learning are capability, opportunity, and motivation.^11^ Today’s trainees have unprecedented access to psychopharmacological information about efficiency, organization, detail, and volume. While most programs provide some instruction of how to access, appraise, and apply patient-relevant evidence for the purpose of supporting practice, this instruction typically is not reinforced by clinical supervisors, thereby segregating knowledge and skill from clinical care. Even less well emphasized, if at all, is the expectation to fully examine clinical experiences involving psychotropics. To develop necessary competencies, clinical experiences should be contextualized by routinely examining them by such considerations as are identified in Table 1. External and internal drivers of motivation matter here. The commonplace approach of a superficial review of textbook chapters and practice guidelines is not sufficient.

It must be emphasized that residency experiences (for psychiatrists, family practitioners, or other physicians) do not provide trainees with what they need to know to become, and continue to be, competent practitioners. Psychiatry residents typically complete residency training with a rather short list of extensively used medications, but little experience with other treatments that will be needed routinely in clinical practice. Moreover, with more extensive clinical experience with an expanding mix of patients, even familiar medicines will present novel experiences, such as unfamiliar adverse effects. The challenge of post-residency learning about how to manage novel psychopharmacological problems is that much greater for primary care physicians who receive far less training in psychiatry yet are responsible for a high proportion of prescriptions for psychotropics in the current era.^12^,^13^

## Practice-Based Learning

Given the shift of modern delivery of mental health clinical services in specialized and primary care settings toward brief assessments, infrequent and short follow-up visits, and heavy reliance on the use of psychotropics, it is essential that residency programs optimize the psychopharmacologic knowledge and experiences of their trainees. However, no matter how much effort goes into improving such training, experiential learning will inevitably be constrained by the time available and by competing demands on the trainee’s attention. It is important to acknowledge that training in psychopharmacology is far from complete when a resident transitions to the role of independent practitioner, and that lifelong, ongoing improvement in knowledge and skill is required. Remarkably, however, this topic has received little critical discussion.^14^–^16^ Aside from individual instances of consulting an expert or reading about a new challenge, most of such continuing professional development and education for health professionals takes the form of conferences, workshops, online programs, or other CME activities.^17^,^18^ The effectiveness of such CME activities has been reviewed critically in recent years.^19^–^21^ Methods of continuing professional development and education continue to evolve, but the means of assuring competence are quite limited. Self-test questions are typically a component of continuing education programs, but these are rarely sufficient or appropriate as a measure of practice competence. Moreover, recertification of medical specialists is a relatively recent development in only some countries, and cannot assure competence in all areas.^22^–^24^ Even less satisfactory is reliance on licensing bodies that only address situations of egregious negligence and incompetence that are brought to their attention. As evidenced below, a system is needed that supports the development and demonstration of minimum competencies for the pharmacological management of people with mental illness.

## Evidence of Poor Pharmacological Management

It is beyond the scope of this brief In Review paper to comprehensively examine competency in the pharmacological management of mental illness. Here, several examples are used to underscore the concerns raised in this and in the In Review paper by Dr Vázquez^25^ and the Guest Editorial by Dr Baldessarini.^26^

During the past 2 decades, use of safer, modern psychotropics has expanded remarkably, as nonpharmacological aspects of assessment and clinical care have declined sharply in the treatment of many types of mental illness. Such trends are particularly clear in the example of MDD.^27^ After receiving a diagnosis of MDD and being prescribed an antidepressant, fewer than one-third of new depressed patients, very often previously unknown to their prescribing clinicians, are followed-up soon and regularly, in accordance with Food and Drug Administration recommendations.^3^,^4^

Many applications of psychotropics continue despite a lack of evidence of either effectiveness for the indication or of safety. For example, use of antipsychotics remains high in vulnerable elders, despite evidence of life-threatening hazards and little evidence of therapeutic benefit in dementias.^28^,^29^ Combinations of antipsychotics, although a routine clinical practice in many centres, provides little, if any, benefit but increases costs and the risk for harm.^30^,^31^ Diagnosis of ADHD and questionable treatment with stimulants is climbing.^32^ Off-label and mostly off-evidence use of antipsychotics in children also has exploded, especially in those diagnosed with ADHD, despite growing evidence of medical risks involved and a lack of adequate safety data for nonpsychotic juveniles.^33^–^39^ There also are concerns about the evident overdiagnosis of bipolar disorder in children, and associated, aggressive use of anticonvulsants and antipsychotics, with largely unproved long-term mood stabilizing effects and little testing in this population.^40^ Although its use in the treatment of schizophrenia has declined, quetiapine now represents one-half of all antipsychotic prescriptions in Canada, with rapidly increasing use in mood and anxiety disorders as well as insomnia, especially in moderate doses.^41^ The abuse potential of this agent and its overprescription for prison inmates further exemplify prescribing and patient care inadequacies.^42^,^43^ From 1995 to 2003, antidepressant use during pregnancy increased 5-fold, only to fall precipitously following regulatory warnings of perinatal complications.^44^ In contrast, exposure during pregnancy to antpsychotics and their combination with other psychotropics, including potentially teratogenic anticonvulsants, is increasing.^45^

These and other inconsistencies between prevalent clinical practices and research findings as well as regulatory warnings have led to growing, largely adverse, reporting on psychotropics in the news media. Such negative publicity about psychotropics contributes to public perceptions that they are to be avoided for being overprescribed, harmful, and limited in benefits.^27^,^46^,^47^ Other reports indicating enmeshment of psychiatry with the pharmaceutical industry add to public concern about the proliferation of psychotropics.^48^,^49^

It is in this context that we recommend psychotropics to patients. To do so competently means having an adequate grasp of findings from clinical research on the effectiveness and safety of specific pharmaceutical products, as well as considerable sensitivity to the opinions and perspectives of individual patients. Therapeutic success requires respect for patients’ preferences, and interest in understanding and clarifying their fears and apprehensions about proposed treatments. However, too often, patients arrive at the pharmacy ambivalent about their diagnosis and treatment plan (if given either), with misgivings about what is prescribed.^50^,^51^ The pharmacist can help to rectify a patient’s cognitive dissonance but is not always successful in doing so.^50^

A simple but informative clinical study in the 1990s reported reasons and timing for prematurely terminating antidepressant treatment.^52^ The leading reasons and median time to discontinuation were as follows: lack of immediate benefit (1 week), adverse effects (4.5 weeks), feeling better (6 weeks), and fear of becoming dependent (8 weeks). Such findings indicate that premature interruption of treatment can be limited or avoided by efforts to assess and communicate with patients initially and repeatedly.^51^,^53^ That is, the skill of the clinician may be more important than the properties of the treatment. Sufficient efforts to enhance acceptance and adherence usually can be time-limited and very practical.^54^,^55^

## Competency Motivation Through Advocacy

Concerns about psychotropics, and about mental health care in general, are prominent topics of discussion in patient self-support groups, at which pressing issues affecting the lives of patients with mental illnesses and their families quickly emerge. Experiences with such groups have been very informative to me, identifying the concerns of patients and families that I may otherwise overlook and need to learn more fully. They are also a reminder that standard, ongoing clinical practice often does not meet the needs of many patients, resulting in the need for additional education and support for patients and families. Themes and issues affecting patients and families that consistently arise at self-help meetings include the following: a perceived wide range of competence, knowledge, and willingness to communicate among clinical professionals of all disciplines; lack of awareness of the purposes or an overall plan behind recommended treatments; frustration with a seemingly idiosyncratic and random approach to selecting and combining psychotropics; lack of recognition and attention to adverse effects; not knowing when to accept a current regimen or to demand a change; and timely access to appropriate care and discussion with prescribers.

## Conclusions

The knowledge and skills needed to use psychotropics safely are incompletely developed when a trainee becomes an independent practising clinician. This truism applies to psychiatrists, primary care physicians, nurse practitioners, other prescribers, and pharmacists. A large proportion of the general public appears to consider psychotropics to be limited in benefits, potentially harmful, and best avoided. At best, public opinion among patients and their families seems to be mixed: psychotropics are seen as necessary and people are hopeful but cautious and often disappointed. Increased efforts are needed to enhance clinical training and knowledge in psychopharmacology among trainees and practising clinicians, with more comprehensive and sustained attention to the assessment of individual patients, and greater reliance on patient education and collaboration.

## Figures and Tables

**Table 1:** Knowledge and skills required for competent prescribing of psychotropics

Knowledge	

Pharmacology	Dosing
Pharmacokinetics	Toxicities
Evidence of effectiveness	Effective alternatives
Gaps in the clinical research base	Access
Adverse effects and their management	Cost
Drug interactions	

Practice skills	

Considerations in special populations	Assessment of
Social context	• Self-care and decision-making style
Barriers to care	• Response
Communications for	• Tolerance and safety
• Developing and modifying care plans	• Adherence
• Informed consent	• Ongoing treatment consent
• Setting treatment expectations	Patient rights
• Evaluating risks over time	

## Abbreviations

ADHD: attention-deficit hyperactivity disorder
CME: continuing medical education
MDD: major depressive disorder
TCA: tricyclic antidepressant
